# Bibliometric analysis of publications that cited the CIOMS 2016 “International ethical guidelines for health-related research involving humans”

**DOI:** 10.1016/j.heliyon.2024.e36833

**Published:** 2024-08-30

**Authors:** Robin Haunschild, Joanne Kays, Lembit Rägo, Mark Kays

**Affiliations:** a Max Planck Institute for Solid State Research, Heisenbergstr. 1, 70569, Stuttgart, Germany; b Joanne S. Kays Consulting, Carmel, Indiana, USA; c CIOMS, Geneva, Switzerland; d Interclarity, LLC, Carmel, Indiana, USA

**Keywords:** Bibliometrics, CIOMS, Ethics, Informed consent, Research ethics, Clinical trials, Children, Women, Adolescents

## Abstract

The CIOMS book “International Ethical Guidelines for Health-related Research Involving Humans”, published in 2016 (IEG2016), provides information to assist research ethics committee members and research practitioners with pragmatically implementing ethical considerations while planning and conducting their research. To identify which aspects of research IEG2016 has had the greatest impact since its publication, we analyzed metadata from 942 papers that cited IEG2016 (English language title only) from Web of Science (WoS, Clarivate). Using VOSviewer, we mapped the co-occurrence of keywords to derive the network of all keywords that co-occurred at least five times in the set of citing papers. We found that the keywords *ethics*, *research ethics*, *informed consent*, and *clinical trials* had high co-occurrence scores in this set of publications. Strong links were also observed between *ethics*, *research ethics*, and *informed consent*. We identified fifteen human-related (HR) keyword nodes in this keyword network. Analysis of the subset of 273 IEG2016-citing articles containing these fifteen HR keywords showed later-date publications were focused on the youngest humans (*children*, *adolescents*, *young people*, *minors*) and the humans typically responsible for those youngest humans, namely *women* and *parents*. Seventy-nine of the 110 networked countries/regions associated with IEG2016-citing articles were home to HR keyword articles. We conclude that IEG2016 has had significant impact in health and medical science literature and has served as a foundation for health-related research around the world in the areas of *ethics*, *informed consent*, and *research ethics* and the linkage of these topics to under-represented populations in such research.

## 1 Introduction

The Council for International Organizations of Medical Sciences (CIOMS) is an international non-governmental, non-profit organization in official relations with the World Health Organization (WHO) and is an associate partner of the United Nations Educational, Scientific, and Cultural Organization (UNESCO). Founded in 1949, it currently includes 40 international, national, and associate member organizations. The mission of CIOMS is to advance public health through guidance on health research and policy including ethics, medical product development, and safety [1].

Historically, CIOMS has been dealing with a wide range of ethical issues and has issued recommendations that were pioneering and innovative at the time (e.g., on protection of prisoners against torture, medical genetics, research involving animals and clinical research involving humans) [2]. The aim of CIOMS ethical guidelines for research in humans has always been to provide internationally vetted ethical principles together with detailed commentary on how these principles should be applied, with particular attention to low-resource settings. This pragmatic focus on feasibility in difficult circumstances has been appreciated, and the guidelines have been widely used around the world, including in low- and middle-income countries (LMICs) [[3], [4], [5], [6]].

In 2016, CIOMS published the “International Ethical Guidelines for Health-related Research Involving Humans” (IEG2016) – a document that combines the topics of two earlier CIOMS guidelines publications to cover both biomedical research and epidemiological studies including biobanking and research with health-related data [7]. The IEG2016 was developed in collaboration between CIOMS and the World Health Organization (WHO), and in close cooperation with the World Medical Association (WMA). They are based on other authoritative ethical guidance documents, including the WMA's Declaration of Helsinki [8] and UNESCO's Universal Declaration on Bioethics and Human Rights [9], and also consider other documents from UNESCO, WHO, the Council of Europe, as well as various regional and international initiatives that had emerged or changed at the time when the guidelines were drafted [10].

The IEG2016 consists of 25 numbered guidelines on specific topics. Each of these starts with the core principles, followed by extensive, carefully worded commentaries with general considerations, justifications, and conditions of their application. They complement the Declaration of Helsinki, facilitating its implementation. The annexes to the CIOMS guidelines have practical tools, e.g., a list of items to be included in research protocols, and essential information to be provided to prospective participants for their informed consent.

With publication of the IEG2016, CIOMS’ aim is to provide internationally vetted ethical principles combined with detailed commentaries on how these universal ethical principles should be applied in practice. The intended outcome is high impact: a contemporary, practical set of basic principles covering key ethical considerations that will be widely used by key audiences (including ethics committees, researchers, academics, patient organizations and regulatory authorities) in health research. It has been widely accepted that CIOMS guidelines manage to strike a balance between the protection of human participants in health-related research and the promotion of such research activities in an exemplary way [10,11].

To our knowledge there has not been much research trying to assess the impact of ethical guidelines. We are also not aware of any internationally accepted indicators to measure impact of ethical guidelines. We propose that one indicator of impact of ethical guidelines, among others, is the extent to which the document is cited in academic papers. Using raw citation counts is problematic. Normalization of citation counts is not feasible because there is no database of ethical guidelines with their citation counts. Documents that state ethical guidelines are usually not covered by citation databases. Thus, we propose to contextualize the citation information with topics (i.e., keywords from citing papers), authors, their institutions, and countries/regions. More indirect indicators of impact such as the number of languages into which the document is translated also exist. By today the IEG2016 has been translated into all six official UN languages (Arabic, Chinese, English, French, Russian, and Spanish) plus Japanese, Korean, Polish, Portuguese, and Ukrainian. The current research project focused only on publications that cited the English version.

## 2 Methodology and dataset

### 2.1 Dataset

We used data from Web of Science (WoS) [12] provided by Clarivate. The IEG2016 is not indexed in WoS. We did not find the IEG2016 as a source item in any other major citation database, either. Thus, we extracted all metadata (with their cited references) from all papers citing the IEG2016 from the web interface of WoS (https://login.webofknowledge.com) via the cited references search with the English title. The following WoS indices were used: SCI-E, SSCI, AHCI, CPCI-S, CPCI-SSH, BKCI-S, BKCI-SSH, ESCI, CCR-E, and IC. However, the latter two did not contribute to the result set. We searched the cited years 2016–2023 for the full title as cited work and cited title. We also searched for the abbreviated version “Int Ethical Guidelin” as cited work during the same time period. In total, 942 papers were downloaded from Web of Science (WoS) on September 05, 2023. As the IEG2016 contains guidelines for health-related research involving humans, this dataset was further refined to a 273-paper dataset which only included papers with at least one of 15 HR keywords (*adolescents*, *adults*, *children*, *community*, *minors*, *parents*, *participants*, *people*, *populations*, *pregnant women*, *research participants*, *researchers*, *vulnerable populations*, *women*, and *young people*). We retrieved these 15 HR keywords from the dataset of all IEG2016 citing papers. To avoid confusion, we took the keywords verbatim and did not attempt to merge keywords that might appear to overlap (e.g., *minors* and *adolescents*). We restricted the search for the English book title because WoS has a bias in indexing towards English literature.

### 2.2 Methods

#### 2.2.1 Co-occurrence maps

We used VOSviewer [13] to map the co-occurrence of keywords (author keywords and keywords plus), authors, affiliations, and countries/regions of authors of the papers citing the book. WoS splits some countries into regions, e.g., the United Kingdom appears as England, Scotland, Wales, and Northern Ireland. We did not merge such splits to avoid discussions about sensitive issues. Network keyword nodes use some basic unification of obvious synonyms (e.g., clinical-trials replaced by clinical trials; risk replaced by risks; etc.; see Supplemental Table S1). The distance between two nodes is determined by the co-occurrence frequency of the terms. The size of the nodes is dependent on the number of papers with a specific keyword, co-author, affiliation, or author country/region. Nodes can be colored according to various characteristics (e.g., cluster assignments, or average publication year). In the results and discussion below, the “average link strength” of any node was calculated by dividing its total link strength by its number of links. In addition to the static VOSviewer maps, we produced interactive versions via VOSviewer Online [14,15].

## 3 Results

**Fig. 1:**
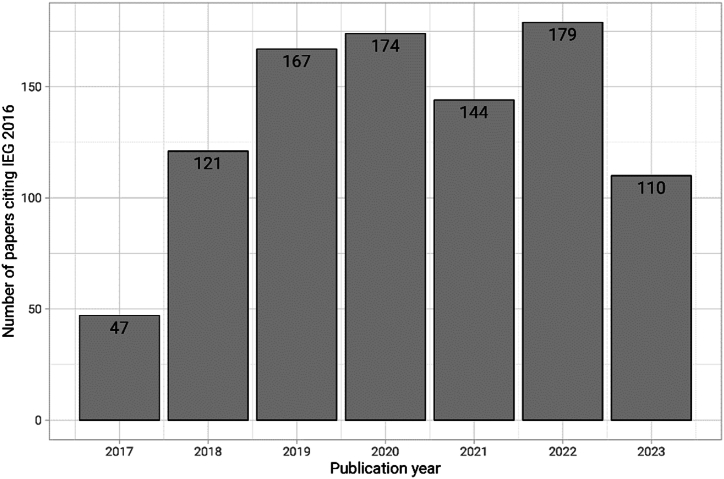
Distribution of IEG2016-citing publications from 2017 through September 5, 2023.

Fig. 1 shows the annual citation profile of IEG2016. There was a bi-phasic distribution of all 942 publications citing IEG2016 from 2017 to 2023. Steady growth in IEG2016-citing articles was seen between 2017 and 2020, followed by a decrease in 2021 and a second and larger peak in 2022. The decline seen in 2023 may be partly explained by having an incomplete year of data (Fig. 1).

Nearly 70 % (n = 652) of the papers citing the book are articles; the remainder is distributed across the following document types: review (n = 114), editorial material (n = 83), book chapter (n = 36), early access (n = 36), letter (n = 14), and proceedings paper (n = 7). There were 285 unique journals that published at least one IEG2016-citing article. The list of journals with at least ten IEG2016-citing articles is included in Supplemental Table S2.

**Fig. 2:**
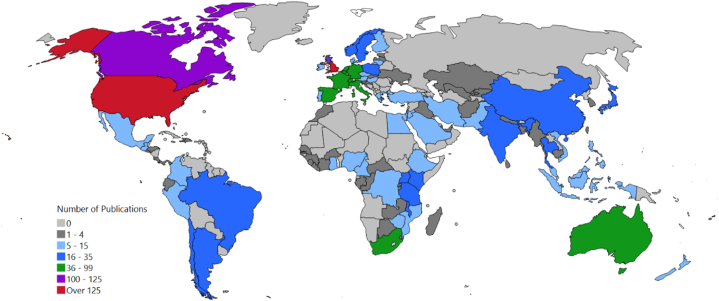
World map of co-publication volumes in countries/regions with IEG2016-citing publications. Derived from the country/region co-publication network (giant component) of all papers citing the IEG2016 (Supplemental Fig. S1). An interactive version of the country/region co-publication network is available at the following URL: https://s.gwdg.de/bcnZ8q.

Country/region co-publication network analysis determined the global distribution of articles citing IEG2016 (Supplemental Fig. S1). Co-publication volume categories of the 110 countries/regions with IEG2016-citing articles are depicted in Fig. 2.

### 3.1 Keyword analysis

**Fig. 3:**
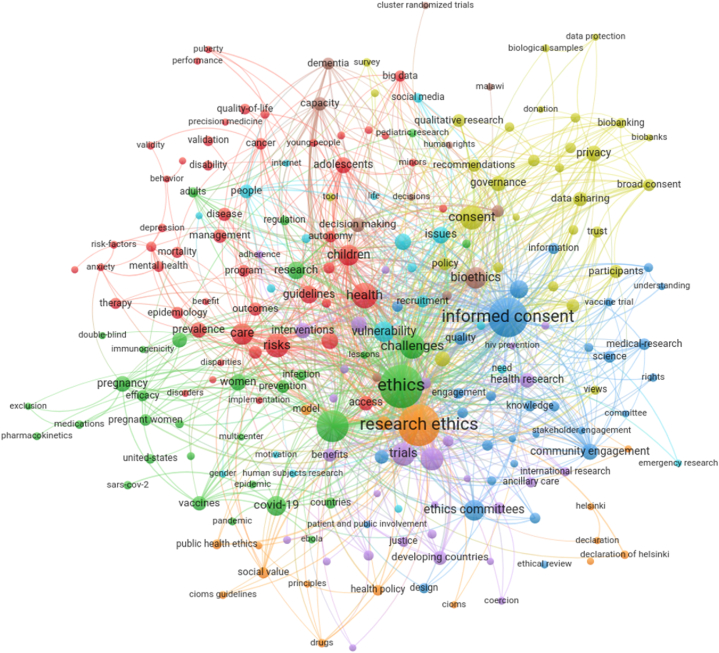
Co-occurrence network of all keywords that co-occur at least five times in the set of citing papers. Link to the interactive version: https://s.gwdg.de/u6Irkz.

Keyword analysis produced 217 nodes (Fig. 3), with 54 nodes comprising the top quartile (Q1) of network nodes (according to average link strength in Table 1 and co-occurrences in Table 2) including 35 nodes having an average link strength greater than the overall average link strength (1.753). *Ethics*, *informed consent*, and *research ethics* were the three top nodes possessing both the keywords' highest average link strength (Table 1) and the keywords’ highest co-occurrences (Table 2).

**Table 1:** Q1 keyword nodes ranked by average link strength (HR keywords highlighted).

Rank	Node	Average link strength
1	Ethics	4.31
2	Informed Consent	4.18
3	Research Ethics	4.12
4	Clinical Trials	2.82
5	Challenges	2.42
6	Risks	2.32
7	Vulnerability	2.25
8	Clinical Research	2.22
9	Trials	2.20
10	Community Engagement	2.14
11	Health	2.14
12	Puberty	2.14
13	Consent	2.12
14	Bioethics	2.10
15	Decision Making	2.08
16	Broad Consent	2.05
17	Participation	2.02
18	Ethics Committees	2.01
19	Care	2.01
20	**Children**	1.99
21	Pregnancy	1.96
22	Privacy	1.94
23	**Adolescents**	1.94
24	Research	1.94
25	Capacity	1.92
26	Dementia	1.92
27	Issues	1.89
28	Developing Countries	1.88
29	**People**	1.85
30	Declaration Of Helsinki	1.83
31	HIV	1.83
32	**Women**	1.82
33	Assent	1.80
34	CoViD-19	1.80
35	Vaccines	1.78
36	Governance	1.75
37	Biobanking	1.72
38	Medical-Research	1.71
39	Health Research	1.69
40	Safety	1.69
41	Data Sharing	1.68
42	Social Value	1.68
43	**Research Participants**	1.66
44	Interventions	1.65
45	Design	1.65
46	Health-Care	1.65
47	Quality	1.64
48	Attitudes	1.63
49	Drugs	1.63
50	Health Policy	1.63
51	Qualitative Research	1.62
52	**Parents**	1.61
53	Recruitment	1.60
54	**Participants**	1.60

**Table 2:** Q1 keyword nodes ranked by co-occurrences (HR keywords highlighted).

Rank	Keyword	Co-occurrences
1	Ethics	179
2	Research Ethics	168
3	Informed Consent	151
4	Clinical Trials	99
5	Health	63
6	Challenges	60
7	Consent	57
8	Risks	54
9	Trials	52
10	Clinical Research	50
11	Bioethics	45
12	Care	45
13	**Children**	42
14	Ethics Committees	42
15	Vulnerability	41
16	CoViD-19	38
17	Participation	32
18	Guidelines	31
19	Research	30
20	Issues	27
21	Public Health	27
22	Health-Care	26
23	**Adolescents**	25
24	Community Engagement	25
25	**Women**	25
26	HIV	24
27	Pregnancy	24
28	Privacy	24
29	Interventions	23
30	Prevalence	23
31	Experiences	22
32	Governance	22
33	Access	21
34	Health Research	21
35	Safety	21
36	Data Sharing	20
37	Decision Making	20
38	Vaccines	20
39	Developing Countries	19
40	Biomedical Research	18
41	Capacity	18
42	Framework	18
43	Management	18
44	**People**	18
45	Benefits	17
46	Disease	17
47	Outcomes	17
48	**Participants**	17
49	Quality	17
50	Africa	16
51	Impact	16
52	Policy	16
53	Prevention	16
54	Qualitative Research	16

### 3.2 HR keyword analysis

The 217 keywords formed the basis for a network with 4372 pairs of linked nodes. The keyword pair with the highest link strength was *informed consent*-*research ethics*, followed by *ethics*-*informed consent* (Table 3). Other nodes in the top ten pairs of nodes ranked by link strength were *clinical trials*, *trials*, *challenges*, *vulnerability*, and *risks*.

**Table 3:** Top ten keyword pairs ranked by link strength.

Rank	Keyword Pair		Link strength
1	Informed Consent	Research Ethics	50
2	Ethics	Informed Consent	38
3	Clinical Trials	Ethics	30
4	Ethics	Research Ethics	29
5	Clinical Trials	Informed Consent	25
6	Informed Consent	Trials	25
7	Challenges	Ethics	24
8	Clinical Trials	Research Ethics	24
9	Research Ethics	Vulnerability	21
10	Ethics	Risks	19

Within the 217 keyword nodes, nine of the fifteen HR keyword nodes describe specific types of humans; the other six HR keyword nodes refer to more general categories (Table 4). Four specific HR keyword nodes were in the top quartile of nodes ranked by average link strength: *children*, *adolescents*, *women*, and *parents* (Table 1). Interestingly, *children*, *adolescents*, and *women* were also in the top quartile when ranking by the number of co-occurrences (Table 2).

**Table 4:** HR keyword nodes grouped into specific and general, each sorted alphabetically.

Specific	General
Adolescents	Community
Adults	Participants
Children	People
Minors	Populations
Parents	Research Participants
Pregnant Women	Vulnerable Populations
Researchers	
Women	
Young People	

The most frequent pairings of an HR keyword with a non-HR keyword were *children*-*informed consent*, *adolescents*-*informed consent* and *informed consent*-*people*. The HR keyword *women* was predominantly paired with four non-HR keywords: *ethics*, *pregnancy*, *research ethics*, and *risks* (Table 5).

**Table 5:** Highest HR keyword to non-HR keyword pairs ordered by link strength.

Keyword Pair		Link strength
Children	Informed Consent	13
Adolescents	Informed Consent	7
People	Informed Consent	7
Parents	Informed Consent	6
Populations	Vulnerability	6
Pregnant Women	Research Ethics	6
Women	Ethics	6
Women	Pregnancy	6
Women	Research Ethics	6
Women	Risks	6
Participants	Ethics	5
Research Participants	Clinical Trials	5
Vulnerable Populations	Research Ethics	5
Adults	Dementia	4
Participation	Community	4
Minors	Consent	4
Young-People	Autonomy	3
Researchers	Risks	2
Researchers	Informed Consent	2

**Fig. 4:**
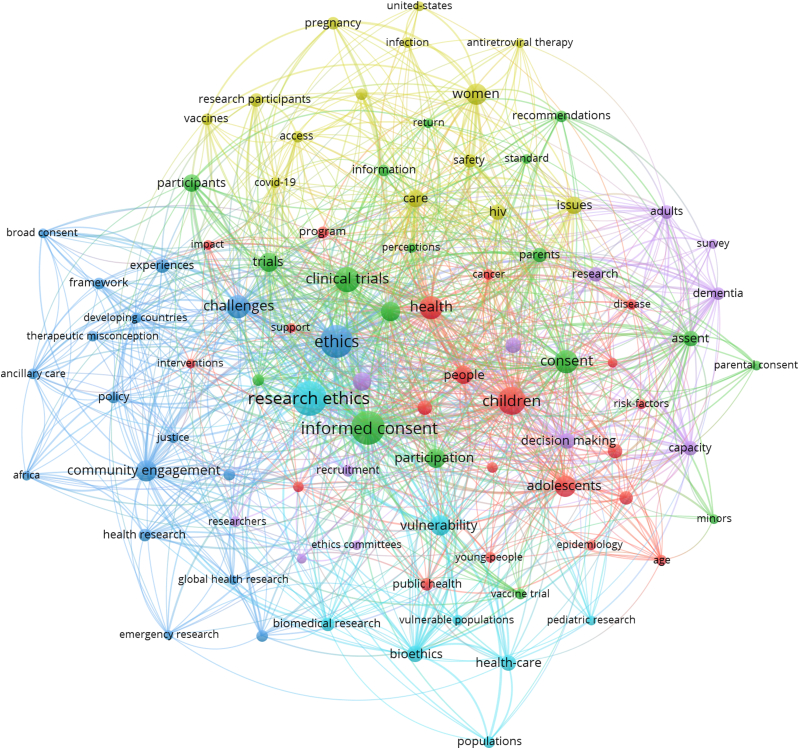
All keywords that occur at least five times in the publication set that contain HR keywords. The nodes are colored according to cluster assignment. Link to the interactive version: https://s.gwdg.de/FzKAXP.

The keyword network refined to the articles containing HR keywords is shown in Fig. 4. There were 273 HR-keyword-containing articles which resulted in 90 keyword nodes co-occurring at least five times within this refined dataset. Of the 273 HR-keyword-containing articles, 193 included only a single HR keyword; 55 had two HR keywords; 23 had three HR keywords; two had four HR keywords. Within this HR-keyword-containing subset, *ethics*, *informed consent*, and *research ethics* remained dominant nodes (see Table 6, Table 7).

**Table 6:** Q1 keyword nodes of articles containing HR keywords ranked by average link strength.

Rank	Keyword	Average link strength
1	Informed Consent	3.99
2	Ethics	3.68
3	Research Ethics	3.31
4	Community Engagement	2.46
5	Children	2.38
6	Clinical Trials	2.37
7	Challenges	2.28
8	Vulnerability	2.26
9	Adolescents	2.24
10	Women	2.16
11	Consent	2.15
12	People	2.14
13	Health	2.14
14	Participation	2.07
15	Assent	2.06
16	Capacity	2.03
17	Decision Making	2.02
18	Clinical Research	1.98
19	Bioethics	1.97
20	Care	1.96
21	Trials	1.93
22	Participants	1.93
23	Issues	1.92

**Table 7:** Q1 keyword nodes of articles containing HR keywords ranked by co-occurrences.

Rank	Keyword	Co-occurrences
1	Research Ethics	65
2	Informed Consent	62
3	Ethics	58
4	Children	42
5	Clinical Trials	34
6	Health	30
7	Challenges	29
8	Consent	28
9	Adolescents	25
10	Community Engagement	25
11	Women	25
12	Vulnerability	23
13	Trials	21
14	Clinical Research	20
15	Participation	20
16	People	18
17	Risks	18
18	Care	17
19	Participants	17
20	Issues	16
21	Bioethics	15
22	Decision Making	14
23	Assent/Health-Care/HIV/Pregnant Women	13

**Fig. 5:**
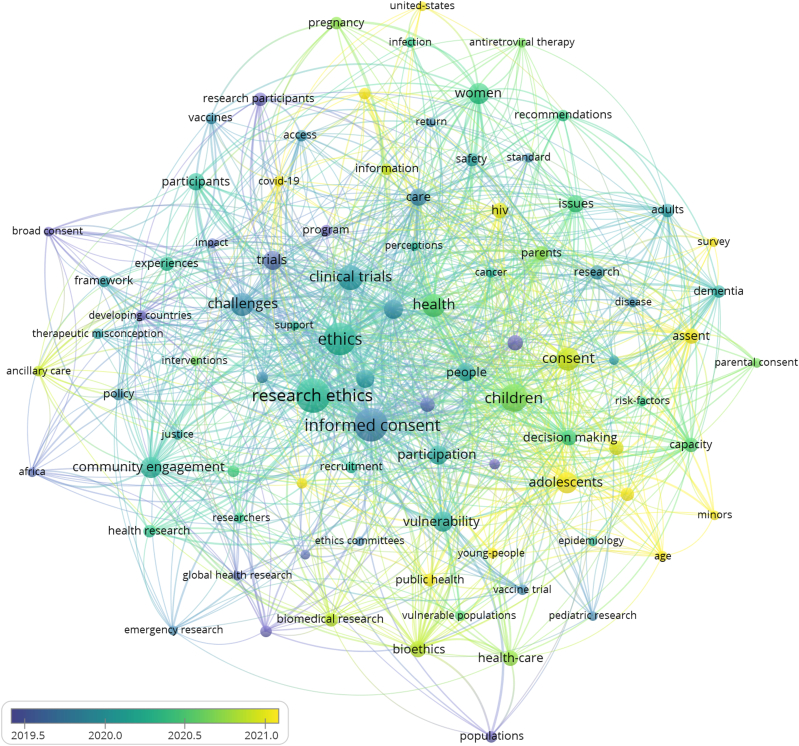
Network of all keywords that occur at least five times in the publication set that contain HR keywords. The nodes are colored according to the average publication year of the corresponding articles. Link to the interactive version: https://s.gwdg.de/NQq55N.

**Fig. 6:**
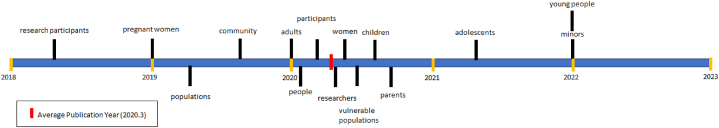
Timeline of average publication years of HR keyword nodes. The average publication year of all 273 HR keyword articles is 2020.3, designated by the red bar on the timeline. (For interpretation of the references to color in this figure legend, the reader is referred to the Web version of this article.)

Fig. 5 shows the keyword network from articles containing HR keywords with node coloring by average publication year. Citing papers that bear HR keywords had an average publication year of 2020.3. Seven of the fifteen HR keywords had an average publication year prior to 2020.3 (Fig. 6). Of the seven, two were specific HR keywords. Of the eight HR keywords published after 2020.3, seven were specific. *Adolescents*, *minors*, and *young people* were the specific HR keyword nodes published most recently.

**Fig. 7:**
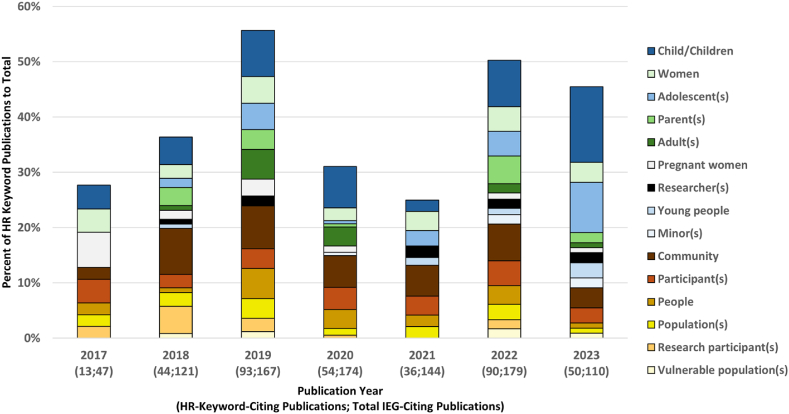
HR keyword publications by year (as percentage of all IEG-citing publications)

The distribution of the percentage of HR keyword papers per year (Fig. 7) was biphasic with a peak of 56 % in 2019, a trough of 25 % in 2021 which then increased to 50 % in 2022. Except for 2018 and 2021, there were more citing papers that bear specific HR keywords than general HR keywords (Fig. 7 and Supplementary Table S3). Since the trough of HR keyword articles in 2021, it appears that there was a greater focus on publishing articles with specific HR keywords.

During the analyzed time period, the top three specific HR keywords in terms of numbers of articles that cited IEG2016 were *children*, *women*, and *adolescents*. *Children* and *women* were the only two of the nine specific HR keyword nodes which had at least one publication in each of the years examined; this was true for four of the six general HR keywords (*community*, *participant*, *people*, and *population*). The publication year 2019 had the highest number of HR keyword publications (n = 93), while 2022 was the only year that had at least one publication for each of the 15 HR keywords (Supplemental Table S3).

### 3.3 Author analysis

**Fig. 8:**
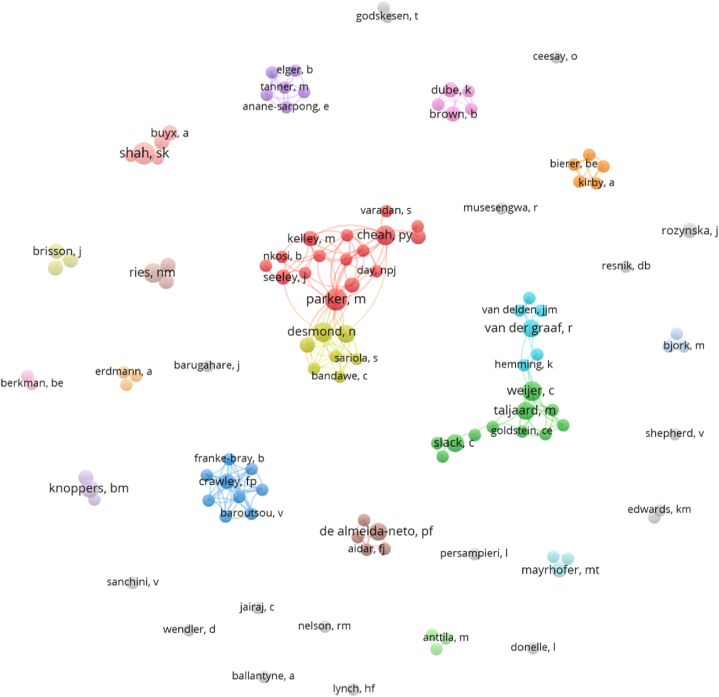
Co-authorship network of co-authors that appeared at least twice based on the HR keyword publication set (disconnected nodes are included). Link to the interactive version: https://s.gwdg.de/nqiJmY. An interactive version with average publication year coloring of the nodes is available via this link: https://s.gwdg.de/r3RbNq.

Fig. 8 depicts the network of all co-authors that co-occur at least twice in the network of IEG2016-citing HR keyword papers. This network contains 116 authors in 34 clusters with 226 links and a total link strength of 382. Authors with four or more IEG2016-citing documents were spread across eight different author clusters (Table 8). Parker, M (Cluster 1, red) and Shah, SK (Cluster 10, salmon) published the highest number of HR keyword IEG2016-citing articles with six each. There were only five of the 116 authors in the network who participated in the development of IEG2016 (i.e., IEG working group authors): Kurihara, C (Cluster 3, two documents), Rid, A (Cluster 10, three documents), van Delden, JJM (Cluster 6, two documents), van der Graaf, R (Cluster 6, four documents), and Wendler, D (Cluster 34, two documents). Taken together, this sub-group of IEG working group authors accounts for 4.3 % of the HR keyword authors, 4 % of the HR keyword documents, and 8.6 % of the total link strength of the HR keyword author network. These data show that IEG2016 had its main impact outside of the IEG working group. Subsequent testing^1^ [16] provided evidence that authors who participated in the development of IEG2016 did not have an impact significantly different than other authors in terms of production (number of documents, p = 0.4676) or collaborations (number of links; p = 0.4904 or average link strength; p = 0.4173).

**Table 8:** Authors of at least four IEG2016-citing papers with at least one HR keyword.

Rank	Author	Cluster	Documents	Avg. pub. year
1	Parker, M	1	6	2019.00
2	Shah, SK	10	6	2020.17
3	Desmond, N	4	5	2019.60
4	Weijer, C	2	5	2019.80
5	Cheah, PY	1	5	2020.40
6	Ries, NM	17	5	2020.20
7	Van Der Graaf, R	6	4	2019.50
8	Nyirenda, D	4	4	2020.75
9	Taljaard, M	2	4	2020.00
10	Slack, C	2	4	2020.75
11	Knoppers, BM	14	4	2020.75
12	De Almeida-Neto, PF	8	4	2022.75

### 3.4 Institutional analysis

**Fig. 9:**
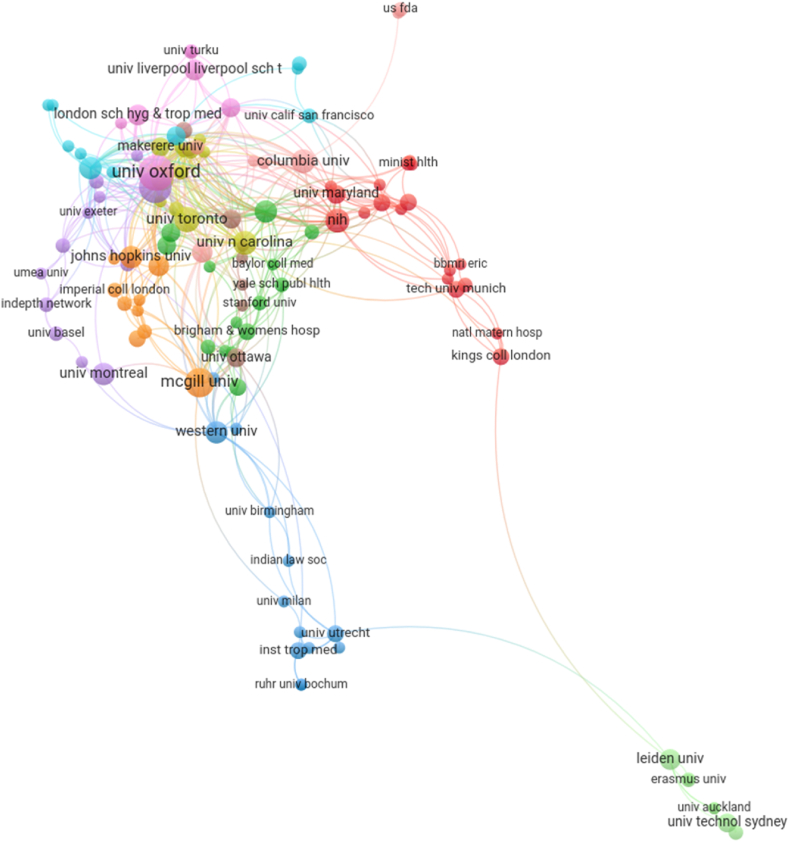
Institutional network based on the HR keyword publication set with at least two papers per institution. Only the connected nodes (giant component) are shown. Link to the interactive version: https://s.gwdg.de/sFuQu4. An interactive version with average publication year coloring of the nodes is available via this link: https://s.gwdg.de/UdOhpc. The node closest to "univ oxford" is "univ kwazulu natal".

Fig. 9 shows the institution network based on the HR keyword publication set. This network contains 119 institutions in 11 clusters with 482 links and a total link strength of 549.

Oxford University had the highest number of co-published documents associated with HR keywords. The University of KwaZulu-Natal ranked second, representing the only institution from an LMIC^2^ in the top ten (Supplemental Table S4). Additional institutions from LMICs in the first quartile ranking by number of documents were the University of Witwatersrand, Mahidol University, Makerere University, the University of Cape Town, and the University of Malawi.

Oxford University had the two highest link strengths with Mahidol University and University Liverpool School of Tropical Medicine, respectively. Of the nine institution-pairings that had link strengths of three or higher, Oxford University had two additional links, with the University of KwaZulu-Natal and the University of Malawi (Supplemental Table S5).

The University of KwaZulu-Natal had the highest number of links (36) and highest link strength (50) of all institutions. Oxford University followed with 29 links and a total link strength of 42. Three additional universities from LMICs (The University of Witwatersrand, the University of Cape Town, and the University of Malawi) were in the top ten institutions whether ranked by number of links or by total link strength (Supplemental Table S6).

### 3.5 Country/region analysis

**Fig. 10:**
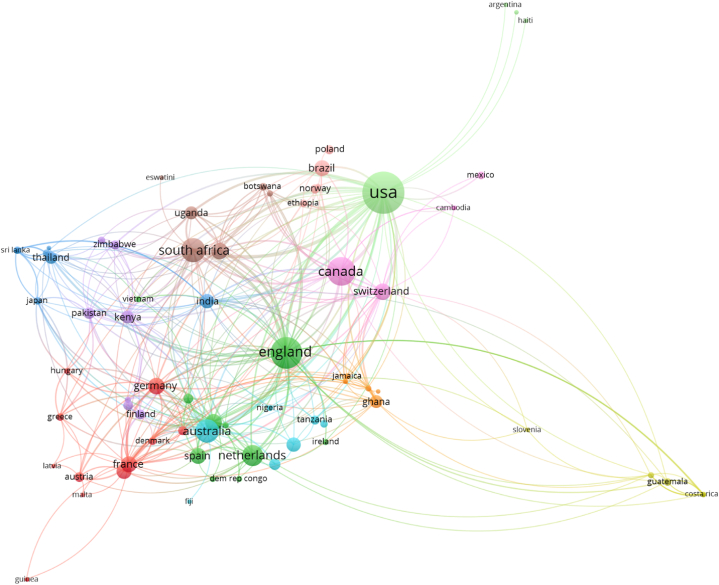
Country/region network based on the HR keyword publication set with at least one paper per country/region. Link to the interactive version: https://s.gwdg.de/lxD25D. An interactive version with publication year coloring of the nodes is available via this link: https://s.gwdg.de/2ZojaH.

The country/region network derived from IEG2016-citing HR keyword papers (Fig. 10) contained 79 countries/regions across 11 clusters with 399 links and a total link strength of 639. The 79 countries/regions were distributed as follows: 35 High-Income Countries (HICs), 38 Middle-Income Countries, and 6 Low-Income Countries (i.e., 44 LMICs). The 399 links (pairings) between countries/regions were distributed as follows: 136 were between two HICs, 165 were between one HIC and one LMIC, and the remaining 98 were between two LMICs. Cluster 8 was the only cluster represented entirely by LMICs. This underlines the high impact of IEG2016 on research from LMICs and collaborations between HICs and LMICs. A complete list of country-cluster members is included in Supplemental Table S7.

The USA was the country/region possessing the highest number of co-published documents. South Africa and Malawi were the two LMICs in the top ten countries with the highest number of documents (Supplemental Table S8). In addition, LMICs in the top quartile of number of documents included Brazil, India, Ghana, Kenya, Uganda, and Thailand.

The USA had the highest total number of links to other countries/regions with 45 (Supplemental Table S9). England and France were next with 44 and 31, respectively. Nine LMICs were in the top quartile of links to other countries/regions: India, South Africa, Ghana, Malawi, The Philippines, Kenya, Pakistan, Thailand, and Uganda.

The highest link strength (12) between two HICs was between Canada and the USA. The highest link strength (14) between a HIC and LMIC was shared by the USA and South Africa. The two LMICs with the highest link strength (4) were South Africa and Uganda. See Supplemental Table S10 for additional country pairs by income category.

**Fig. 11:**
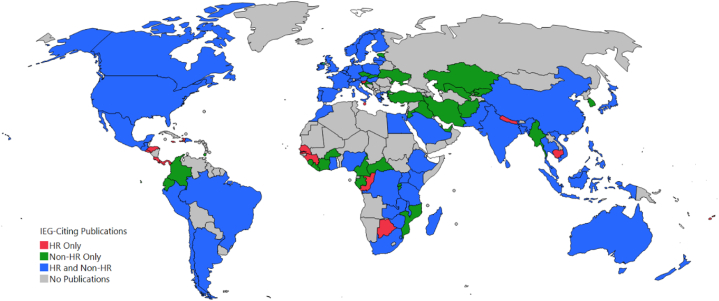
World map of countries specifying the types of IEG2016-citing publications.

Fig. 11 updates the global distribution of IEG2016-citing countries in Fig. 2 by differentiating the presence or absence of HR keywords. Of the 110 networked countries/regions with IEG2016-citing publications, fifteen countries/regions had only HR keyword articles; 31 had only non-HR keyword publications; and the remaining 64 had both HR and non-HR keyword publications.

## 4 Discussion

The purpose of this bibliometric analysis was to use IEG2016 citations as a basis from which to propose a contextualized impact measurement of published ethical guidelines. While developing the proposed methodology, we applied it to the IEG2016. Although the total number of 942 citing publications already indicated a substantial impact, a more detailed analysis measured the specific areas where IEG2016 had its highest impact. We identified emergent themes from keyword networks and how authors, their institutions, and countries/regions that cited IEG2016 were networked. Namely, these emergent themes were related to the keywords *ethics*, *informed consent*, and *research ethics* as well as the intersection of these areas with humans (i.e., HR keywords) that have largely been under-represented in health-related research.

Ethics, informed consent, and research ethics are key concepts and recurring themes in IEG2016. The co-occurrence network of keywords reflected the importance of these three concepts in IEG2016-citing publications with *ethics, informed consent* and *research ethics* being the most frequently occurring overall keywords, whether using the entire set of 942 IEG2016-citing publications or the 273 human-related publication subset.

Examining the details some of the IEG2016-citing publications revealed that the scope of IEG2016's influence regarding ethics, informed consent, and research ethics went beyond the settings typically associated with health-related research involving humans. The concepts of ethics and research ethics as applied to health-related research in IEG2016 provided guidance in other areas as diverse as citizen science [17], social work [18], and online research [19]. IEG2016 has also served as a reference document for authors constructing additional ethics guidelines for specific people or disease states. Huria, Palmer [20] was the most subsequently cited publication in the subset of IEG2016-citing articles that contained HR keywords. This paper developed the CONSIDER (“CONSolIDated critERia”) statement, with the goals of strengthening research praxis and advancing the health outcomes of indigenous peoples. Brown and Sugarman [21] devised a set of guidelines specifically for HIV research, referencing IEG2016. Additionally, online training courses covering research ethics and referencing IEG2016 guidelines have been developed [22,23]. Along these same lines, a current CIOMS Working Group is in the process of harmonizing guidelines for education and training syllabi for healthcare professionals globally, including guidance on structuring online courses [24].

IEG2016 strongly recommends expanding/including under-represented/vulnerable people in not only biomedical research, but also in health-related research. Children, women, and adolescents are among the categories of humans considered under-represented, with IEG2016 devoting specific guidelines for including children and adolescents (Guideline 17) and women (Guidelines 18 and 19) as participants in health-related research. Our analysis provides evidence that IEG2016 has had impact on the inclusion of these under-represented humans in recent health-related research, because *children*, *women* and *adolescents* were the HR keywords with the highest ranking based upon node link strength and number of co-occurrences. Furthermore, there is evidence for the intersection of these particular HR keywords with the highest-ranking overall keywords (*ethics*, *informed consent*, and *research ethics*). *Ethics* and *research ethics* shared equal link strengths to the HR keyword *women* (along with *pregnancy* and *risks*), while *informed consent* was the keyword with the highest link strength to HR keywords *children* and *adolescents*. The intersection of these HR and overall keywords suggests that IEG2016-citing researchers may recognize issues linked to lower inclusion numbers for these under-represented groups and the lack of evidence-based information regarding health-related interventions for these particular people. Other under-represented groups and areas where information for interventions are lagging are found in publications from the most productive authors of IEG2016-citing papers that contain HR keywords and include pregnant women [25], indigenous peoples [26], children, and “decisionally-vulnerable” adults [27], people with dementia [28], and adolescent athletes [29] in research areas like medication use, genomics studies, and general health care.

Designing and conducting health-related research, especially when including under-represented groups, can face multiple challenges within a given local setting. IEG2016 Guideline 7 is devoted to community engagement and its related components, such as the inclusion of minorities or marginalized groups, individual informed consent, dissemination of data and results outside the community, and building confidence and trust between the community and the researchers. These facets of IEG2016 were seen in the publications of several IEG2016-citing authors which described the need to consider the extent and means by which community/stakeholder engagement (CE) is the most beneficial to guiding research ethics committees before, during, and after conducting a study [[30], [31], [32]]. The two overall highest-ranking institutions by our analyses, Oxford University and the University of KwaZulu-Natal, showed that community engagement in resource-limited settings was a prominent topic in their IEG2016-citing publications [[33], [34], [35], [36], [37], [38], [39], [40], [41], [42]]. A publication where these two institutions collaborated (along with others), drew attention to improved models and practices for CE, including research on factors that contribute to “good” CE (e.g., adapting to local cultural norms, working with local gatekeepers, treating community members with respect, etc.) [43]. These two institutions also joined in research that provided recommendations on ethically redressing the evidence gap around HIV and co-infections for pregnant people [44] and calls for researchers and institutions to transition from individualistic models of autonomy and agency to models based upon interdependencies of people, institutions, and research structures [45]. Such publications illustrate how IEG2016 served as a framework that researchers could use to initiate CE and adapt to meet the needs of the communities in which they were engaged.

Inclusion alone, however, may not be enough to address the lack of sufficient evidence-based information for under-represented groups in health-related research. It may be necessary to enhance the efficiency of biomedical trials by implementing new, and potentially controversial, clinical trial designs [46,47]. More efficient designs could, in theory, collect sufficient data from under-represented groups in a shorter period and provide an opportunity to determine if differences in treatment efficacy and safety exist for those groups. Developing guidelines for these new trial designs could represent an area for future work.

IEG2016 intends to be a document that can provide guidance and serve as a common basis for collaborations to expand health-related research globally. However, special considerations must be taken into account when conducting health-related research in low-resource regions. As stated above, creating an environment of trust and co-operation via community/stakeholder engagement is a critical factor in ensuring equipoise with a research study. Implementing IEG2016 in LMIC health-related research revealed other practical needs requiring separate and more-detailed attention. Several IEG2016-citing publications [[48], [49], [50]] addressed those specialized needs. Recognizing the need to establish a more collaborative research environment in LMICs, CIOMS created a working group to establish recommendations specifically for stakeholders [51]. This working group report provides 20 recommendations divided between three key stakeholder groups (government and regulatory authorities, researchers, and international organizations and funders) to assist these groups in developing and implementing health-related research involving humans in LMICs. The above activities suggest that IEG2016 is a globally recognized basis in the evolving process of developing and conducting ethical health-related research.

Our study is not without limitations. Publications used for this analysis may not represent the whole body of works that utilize the principles of IEG2016 because they were not published in English or did not directly cite IEG2016. Publications that did cite IEG2016 but are not indexed in WoS are also not included in our analysis. The 15 HR keywords were extracted verbatim from the keyword nodes found in Fig. 3. In filtering the 942 articles for HR keywords to create the network found in Fig. 4, some articles contained phrases that included more than the verbatim HR keyword, e.g., “community” and “community-driven”. Such phrases were implicitly included in our analysis to avoid limiting the scope of the verbatim HR keywords.

## 5 Conclusions

Our analysis of IEG2016-citing articles presents the impact the IEG2016 has had in a contextualized manner. Our results suggest that the IEG2016 has become a standard reference in the field of medical ethics – providing guidance to researchers in the development of new knowledge for ethical principles in health research that involves human subjects. Impact of IEG2016 has been mainly in the areas related to the keywords *ethics*, *informed consent*, and *research ethics*. Importantly, authors extended the development of ethical principles to human subject groups historically under-represented in health-related studies: *children*, *women*, and *adolescents*. IEG2016 has also made an impact on health research publishing from geographic and economic standpoints. While institutions in high-income countries, such as the US and the UK, have been the most involved in producing publications citing IEG2016, institutions in LMICs (especially South Africa) have, individually or in company with HIC institutions, produced research studies citing IEG2016. Overall, we conclude that over the seven years since its publication, IEG2016 has served as a world-wide foundational work regarding medical ethics for researchers conducting biomedical- and health-related studies involving under-represented human subject groups.

## Data availability statement

All data were downloaded from WoS (https://www.webofscience.com). The authors do not have permission to share data.

## CRediT authorship contribution statement

**Robin Haunschild:** Writing – review & editing, Writing – original draft, Visualization, Supervision, Software, Project administration, Methodology, Investigation, Formal analysis, Data curation. **Joanne Kays:** Writing – review & editing, Writing – original draft, Visualization, Methodology, Investigation, Formal analysis. **Lembit Rägo:** Writing – review & editing, Writing – original draft. **Mark Kays:** Writing – review & editing, Writing – original draft, Visualization, Supervision, Software, Methodology, Investigation, Formal analysis, Conceptualization.

## Declaration of competing interest

The authors declare the following financial interests/personal relationships which may be considered as potential competing interests:Associate Editor for the section Information Sciences of Heliyon: RH.

Employee of CIOMS: LR.

If there are other authors, they declare that they have no known competing financial interests or personal relationships that could have appeared to influence the work reported in this paper.

## Footnotes

1 Wilcoxon-Mann/Whitney test.

2 Low-and-Middle Income Countries, Source: World Bank Country and Lending Groups Data, https://datahelpdesk.worldbank.org/knowledgebase/articles/906519-world-bank-country-and-lending-groups.
