# Supplementary material for: Bibliometric analysis of publications that cited the CIOMS 2016 “International ethical guidelines for health-related research involving humans”
Source: Heliyon. 2024 Aug 30;10(17):e36833. doi: 10.1016/j.heliyon.2024.e36833 (PMC11402149; doi:10.1016/j.heliyon.2024.e36833)
Supplement: Multimedia component 1 [file mmc1.docx]

Appendix


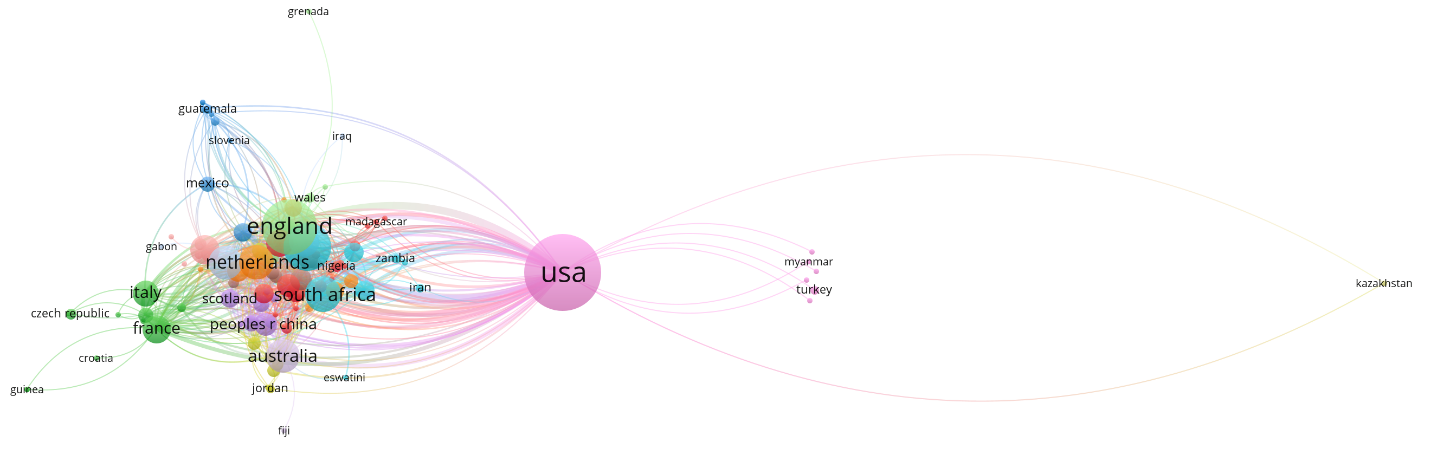


Supplemental Figure S1: Country co-publication network (giant component) of all papers citing the IEG2016. An interactive version is available at the following URL: <https://s.gwdg.de/CyDlxE>

Supplemental Table S1: Obvious synonyms that were unified in creation of the keyword network in Figure 3.

| **Label** | **Replacement** |
| --- | --- |
| biomedical-research | biomedical research |
| challenge | challenges |
| clinical-research | clinical research |
| clinical trial | clinical trials |
| clinical-trials | clinical trials |
| developing-countries | developing countries |
| ethics committee | ethics committees |
| ethics (see medical ethics) | medical ethics |
| research ethics committee | ethics committees |
| research ethics committees | ethics committees |
| experience | experiences |
| informed-consent | informed consent |
| institutional review board | institutional review boards |
| intervention | interventions |
| mental-health | mental health |
| pregnant-women | pregnant women |
| public-health | public health |
| risk | risks |
| trial | trials |
| vaccine | vaccines |

Supplemental Table S2: Journals publishing at least ten IEG2016-citing articles ranked by the number of articles

| Rank | Journal title | Number of articles | Cumulative percent of all articles |
| --- | --- | --- | --- |
| 1 | BMC Med. Ethics | 51 | 5.6% |
| 2 | J. Empir. Res. Hum. Res. Ethics | 38 | 9.9% |
| 3 | Bioethics | 34 | 13.6% |
| 4 | Trop. J. Pharm. Res. | 23 | 16.2% |
| 5 | J. Med. Ethics | 20 | 18.4% |
| 6 | BMJ Open | 18 | 20.4% |
| 7 | Dev. World Bioeth. | 17 | 22.3% |
| 8 | Am. J. Bioeth. | 16 | 24.0% |
| 9 | PLoS One | 16 | 25.8% |
| 10 | Trials | 15 | 27.5% |
| 11 | BMJ Glob. Health | 14 | 29.0% |
| 12 | BioLaw J. | 12 | 30.3% |
| 13 | Account. Res. | 10 | 31.5% |
| 14 | Clin. Trials | 10 | 32.6% |

Supplemental Table S3: Number of publications with HR keywords per year (* denotes specific HR keyword)

| Keyword | 2017 | 2018 | 2019 | 2020 | 2021 | 2022 | 2023 | Total |
| --- | --- | --- | --- | --- | --- | --- | --- | --- |
| Child/Children* | 2 | 6 | 14 | 13 | 3 | 15 | 15 | 68 |
| Women* | 2 | 3 | 8 | 4 | 5 | 8 | 4 | 34 |
| Adolescent(s)* | 0 | 2 | 8 | 1 | 4 | 8 | 10 | 33 |
| Parent(s)* | 0 | 4 | 6 | 1 | 0 | 9 | 2 | 22 |
| Adult(s)* | 0 | 1 | 9 | 6 | 0 | 3 | 1 | 20 |
| Pregnant women* | 3 | 2 | 5 | 2 | 0 | 2 | 1 | 15 |
| Researcher(s)* | 0 | 1 | 3 | 0 | 3 | 3 | 2 | 12 |
| Young people* | 0 | 1 | 0 | 0 | 2 | 2 | 3 | 8 |
| Minor(s)* | 0 | 0 | 0 | 1 | 0 | 3 | 2 | 6 |
| Community | 1 | 10 | 13 | 10 | 8 | 12 | 4 | 58 |
| Participant(s) | 2 | 3 | 6 | 7 | 5 | 8 | 3 | 34 |
| People | 1 | 1 | 9 | 6 | 3 | 6 | 1 | 27 |
| Population(s) | 1 | 3 | 6 | 2 | 3 | 5 | 1 | 21 |
| Research participant(s) | 1 | 6 | 4 | 1 | 0 | 3 | 0 | 15 |
| Vulnerable population(s) | 0 | 1 | 2 | 0 | 0 | 3 | 1 | 7 |
| Totals | 13 | 44 | 93 | 54 | 36 | 90 | 50 | 380 |
| Specific HR keyword | 7 | 20 | 53 | 28 | 17 | 53 | 40 | 218 |
| General HR keyword | 6 | 24 | 40 | 26 | 19 | 37 | 10 | 162 |

Supplemental Table S4: Top ten institutions with at least one HR keyword on each of their IEG2016-citing papers ordered by number of documents

| Institution | Documents |
| --- | --- |
| University of Oxford | 18 |
| University of KwaZulu-Natal | 15 |
| McGill University | 12 |
| University of Toronto | 9 |
| Columbia University | 8 |
| National Institutes of Health | 8 |
| University of North Carolina | 8 |
| Johns Hopkins Bloomberg School of Public Health | 7 |
| Johns Hopkins University | 7 |
| University of Montreal | 7 |

Supplemental Table S5: Institution linked pairs with link strength greater than two ordered by link strength. Only institutions with at least one HR keyword on each of their IEG2016-citing papers are included.

| Institution Pair | | Link Strength |
| --- | --- | --- |
| Mahidol University | University of Oxford | 5 |
| University of Liverpool School of Tropical Medicine | University of Oxford | 4 |
| Columbia University | Harvard Medical School | 3 |
| Johns Hopkins Bloomberg School of Public Health | Johns Hopkins University | 3 |
| University of KwaZulu-Natal | University of Oxford | 3 |
| University of Malawi | University of Oxford | 3 |
| University of Newcastle | University of Technology Sydney | 3 |
| University of Ottawa | Western University | 3 |
| University of Washington | Vanderbilt University | 3 |

Supplemental Table S6: Top ten institutions by number of links. Only institutions with at least one HR keyword on each of their IEG2016-citing papers are included.

| Institution | Links | Total Link Strength |
| --- | --- | --- |
| University of KwaZulu-Natal | 36 | 50 |
| University of Oxford | 29 | 42 |
| University of Washington | 28 | 34 |
| University of North Carolina | 26 | 30 |
| University of Witwatersrand | 23 | 26 |
| University of Cape Town | 22 | 24 |
| Elizabeth Glaser Pediatric AIDS Foundation | 21 | 22 |
| National Institutes of Health | 21 | 24 |
| Columbia University | 20 | 23 |
| University of Malawi | 20 | 23 |

Supplemental Table S7: Countries by cluster from Figure 10 with their assignment to World Bank income groups

| Cluster | Country | Income group |
| --- | --- | --- |
| 1 | Austria | HIC |
|  | Denmark | HIC |
|  | France | HIC |
|  | Germany | HIC |
|  | Greece | HIC |
|  | Guinea | LMIC |
|  | Hungary | HIC |
|  | Italy | HIC |
|  | Latvia | HIC |
|  | Malta | HIC |
|  | Portugal | HIC |
|  | Senegal | LMIC |
| 2 | Belgium | HIC |
|  | Dem Rep Congo | LMIC |
|  | England | HIC |
|  | Gambia | LMIC |
|  | Ireland | HIC |
|  | Luxembourg | HIC |
|  | Netherlands | HIC |
|  | Scotland | HIC |
|  | Spain | HIC |
|  | Vietnam | LMIC |
| 3 | Bangladesh | LMIC |
|  | India | LMIC |
|  | Indonesia | LMIC |
|  | Japan | HIC |
|  | Malaysia | LMIC |
|  | Philippines | LMIC |
|  | Sri Lanka | LMIC |
|  | Taiwan | HIC |
|  | Thailand | LMIC |
| 4 | Costa Rica | LMIC |
|  | Dominican Rep | LMIC |
|  | El Salvador | LMIC |
|  | Guatemala | LMIC |
|  | Honduras | LMIC |
|  | Panama | HIC |
|  | Peru | LMIC |
|  | Slovenia | HIC |
| 5 | Chile | HIC |
|  | Finland | HIC |
|  | Kenya | LMIC |
|  | Pakistan | LMIC |
|  | Peoples R China | LMIC |
|  | Singapore | HIC |
|  | Zimbabwe | LMIC |
| 6 | Australia | HIC |
|  | Fiji | LMIC |
|  | New Zealand | HIC |
|  | Nigeria | LMIC |
|  | Sweden | HIC |
|  | Tanzania | LMIC |
|  | Wales | HIC |
| 7 | Egypt | LMIC |
|  | Ghana | LMIC |
|  | Jamaica | LMIC |
|  | Morocco | LMIC |
|  | Rep Congo | LMIC |
|  | Saudi Arabia | HIC |
| 8 | Botswana | LMIC |
|  | Eswatini | LMIC |
|  | Malawi | LMIC |
|  | South Africa | LMIC |
|  | Uganda | LMIC |
|  | Zambia | LMIC |
| 9 | Cambodia | LMIC |
|  | Canada | HIC |
|  | Madagascar | LMIC |
|  | Mexico | LMIC |
|  | Switzerland | HIC |
| 10 | Brazil | LMIC |
|  | Ethiopia | LMIC |
|  | Lithuania | HIC |
|  | Norway | HIC |
|  | Poland | HIC |
| 11 | Argentina | LMIC |
|  | Haiti | LMIC |
|  | Nepal | LMIC |
|  | US | HIC |

Supplemental Table S8: Top ten countries by documents from Figure 10

| Country | Cluster | Documents |
| --- | --- | --- |
| USA | 11 | 86 |
| England | 2 | 49 |
| Canada | 9 | 39 |
| South Africa | 8 | 28 |
| Australia | 6 | 27 |
| Netherlands | 2 | 22 |
| Belgium | 2 | 14 |
| Germany | 1 | 14 |
| Switzerland | 9 | 14 |
| Malawi | 8 | 13 |

Supplemental Table S9: Top quartile of countries ordered by links to other countries from Figure 10

| Rank | Country | Links |
| --- | --- | --- |
| 1 | USA | 45 |
| 2 | England | 44 |
| 3 | France | 31 |
| 4 | Australia | 29 |
| 5 | Belgium | 28 |
| 6 | Switzerland | 27 |
| 7 | India | 25 |
| 8 | South Africa | 24 |
| 9 | Germany | 22 |
| 10 | Canada | 20 |
| 11 | Ghana | 18 |
| 12 | Netherlands | 18 |
| 13 | Malawi | 17 |
| 14 | Philippines | 17 |
| 15 | Kenya | 16 |
| 16 | Pakistan | 16 |
| 17 | Japan | 14 |
| 18 | Thailand | 14 |
| 19 | Uganda | 14 |
| 20 | Scotland | 13 |

Supplemental Table S10: Country pairings ordered by link strength from Figure 10 grouped by their World Bank income group pairings

| Income pairing | Country pair | | Link strength |
| --- | --- | --- | --- |
| HIC Pairs* | Canada | USA | 12 |
|  | England | USA | 10 |
|  | Switzerland | USA | 8 |
|  | Australia | USA | 7 |
|  | England | Netherlands | 6 |
|  | Australia | Belgium | 4 |
|  | Australia | England | 4 |
|  | Belgium | Germany | 4 |
|  | Canada | England | 4 |
|  | France | Germany | 4 |
| HIC - LMIC Pairs* | USA | South Africa | 14 |
|  | England | Malawi | 8 |
|  | England | South Africa | 8 |
|  | USA | Malawi | 7 |
|  | USA | India | 6 |
|  | USA | Uganda | 6 |
|  | Canada | South Africa | 5 |
|  | England | Thailand | 5 |
|  | England | Kenya | 4 |
|  | USA | Brazil | 4 |
|  | Switzerland | Ghana | 4 |
| LMIC Pairs** | South Africa | Uganda | 4 |
|  | Kenya | South Africa | 3 |
|  | Malawi | South Africa | 3 |
|  | South Africa | Zimbabwe | 3 |

* Link strengths of 4 or higher.

** Link strengths of 3 or higher.
